# Effect of 2-Week Naringin Supplementation on Neurogenesis and BDNF Levels in Ischemia–Reperfusion Model of Rats

**DOI:** 10.1007/s12017-023-08771-0

**Published:** 2024-03-08

**Authors:** Esen Yilmaz, Gozde Acar, Ummugulsum Onal, Ender Erdogan, Abdulkerim Kasim Baltaci, Rasim Mogulkoc

**Affiliations:** 1 Department of Medical Physiology, Selcuk University, 42250 Konya, Turkey; 2 Department of Histology, Selcuk University, 42250 Konya, Turkey

**Keywords:** BDNF, DCX, Flavonoid, Naringin, NeuN, Neurogenesis

## Abstract

Background: Ischemic stroke is the leading cause of mortality and disability worldwide with more than half of survivors living with serious neurological sequelae; thus, it has recently attracted a lot of attention in the field of medical study. Purpose: The aim of this study was to determine the effect of naringin supplementation on neurogenesis and brain-derived neurotrophic factor (BDNF) levels in the brain in experimental brain ischemia–reperfusion. Study design: The research was carried out on 40 male Wistar-type rats (10–12 weeks old) obtained from the Experimental Animals Research and Application Center of Selçuk University. Experimental groups were as follows: (1) Control group, (2) Sham group, (3) Brain ischemia–reperfusion group, (4) Brain ischemia–reperfusion + vehicle group (administered for 14 days), and (5) Brain ischemia–reperfusion + Naringin group (100 mg/kg/day administered for 14 days). Methods: In the ischemia–reperfusion groups, global ischemia was performed in the brain by ligation of the right and left carotid arteries for 30 min. Naringin was administered to experimental animals by intragastric route for 14 days following reperfusion. The training phase of the rotarod test was started 4 days before ischemia–reperfusion, and the test phase together with neurological scoring was performed the day before and 1, 7, and 14 days after the operation. At the end of the experiment, animals were sacrificed, and then hippocampus and frontal cortex tissues were taken from the brain. Double cortin marker (DCX), neuronal nuclear antigen marker (NeuN), and BDNF were evaluated in hippocampus and frontal cortex tissues by Real-Time qPCR analysis and immunohistochemistry methods. Results: While ischemia–reperfusion increased the neurological score values, DCX, NeuN, and BDNF levels decreased significantly after ischemia in the hippocampus and frontal cortex tissues. However, naringin supplementation restored the deterioration to a certain extent. Conclusion: The results of the study show that 2 weeks of naringin supplementation may have protective effects on impaired neurogenesis and BDNF levels after brain ischemia and reperfusion in rats.

## Introduction

Stroke is a disease condition defined as the interruption of blood flow to the brain due to a clot or embolism or the rupture of a blood vessel in the brain, which then leads to neurological disorders (Ojaghihaghighi et al., 2017). Currently, a surgical thrombectomy procedure is performed to apply a thrombolytic agent such as tissue plasminogen activator (tPA) or to mechanically remove the blood clot (thrombus) for the treatment of ischemic stroke (Prabhakaran et al., 2015). However, the timeframe available for these treatments is very narrow, and survivors often exhibit limited functional recovery as well as a high degree of morbidity (Dorado et al., 2014). Therefore, there is an urgent need for new treatment modalities that can lead to better functional recovery and reduced morbidity. To this end, brain repair processes through endogenous neurogenesis after stroke have been a particular subject of research. Ischemic injury triggers low-level endogenous neurogenesis in the subventricular (SVZ) and subgranular (SGZ) zones, followed by the migration of neuroblasts to the injury site (Rahman et al., 2021). Therefore, many studies have been conducted to improve stroke-induced neurogenesis by applying appropriate exogenous factors that play a role in the proliferation, migration, and synaptic integration of neural stem/progenitor cells, and thus in motor and cognitive functional recovery after stroke (Marques et al., 2019). Adult neurogenesis is the process of proliferation and differentiation of neural stem/progenitor into either neurons or glia cells (astrocytes and microglial cells), then migration, maturation, and integration of newly born cells into the previously developed neural network (Bartkowska et al., 2022). Neural stem/progenitor cells and substances required for neurogenesis are found in neurogenic niches in the mammalian brain, including the human brain (Culig et al., 2022). The neurogenic niche refers to the local micro-environment containing neural stem/progenitor cells that are self-renewing and have the potential to differentiate into different cell types (glial cells and neurons) (Accogli et al., 2020). Neurogenesis has been studied much more extensively in two well-known regions, the SGZ of the hippocampal dentate gyrus and the SVZ of the lateral ventricles (Hagg, 2009). The function of newly born neurons is dependent on the region in which they generate, such as newly born granule cells in the SGZ have a role in hippocampal-dependent learning and memory (Terranova et al., 2019), pattern separation (Treves et al., 2008), cognitive flexibility (Garthe et al., 2016), and forgetting (Anacker et al., 2018). Neurogenesis in the hippocampus can be modulated by environmental factors such as social, sensory, motor, and cognitive enrichment, voluntary physical activity, and hippocampal-dependent learning tasks, such as eye-blink conditioning and spatial navigation learning in the Morris water maze test, enhance hippocampal neurogenesis, in contrary factors such as physical and psychosocial stressors and depression cause a reduction in hippocampal neurogenesis. In addition, various endogenous factors such as developmental morphogens, neurotrophic factors, and neurotransmitters have been reported to influence hippocampal neurogenesis. (Lieberwirth et al., 2016). Brain-derived neurotrophic factor (BDNF) is the best-studied neurotrophic factor in concern with adult neurogenesis and stroke-induced neurogenesis (Leal et al., 2017). Both endogenous and exogenous BDNF enhance the proliferation, survival, migration, and maturation of neural stem/progenitor cells in the SVZ and SGZ (Bath et al., 2012; Bekinschtein et al., 2011). Low BDNF levels are associated with an increased risk of stroke, worse functional outcomes, and higher mortality (Kotlega et al., 2017). After induction of ischemic stroke in animals, the elevation of BDNF levels by various endogenous and exogenous factors has been associated with increased neurogenesis and thus learning, memory, and functional improvement (Kim et al., 2014; Ni et al., 2020; Wang et al., 2004).

The administration of flavonoids stimulates neurogenesis via various pathways in the brain under physiological conditions and after ischemic stroke (Calis et al., 2020; Cichon et al., 2020). Naringin, a natural flavonoid extract from grapefruit and orange, is well known for its anti-inflammatory and antioxidant activities (Zhao & Liu, 2021). Recent studies have shown that naringin has a positive effect on neurogenesis and BDNF levels in both physiological and pathological conditions (Gao et al., 2022a).

The present study aimed to determine the effect of 2-week administration of naringin on neurogenesis in focal brain ischemia–reperfusion in rats.

## Materials and Methods

### Animals

This study was carried out in the Experimental Medicine Application and Research Center of Selcuk University and all animal experimental methods and protocols were approved by the Ethics Committee of the same center (dated 31.12.2021, reference number: 2021-55). Forty Wistar male rats (10–12 weeks old) with an average weight of 300–400 g (350 g) were used in this study. Animals had free access to a standard diet and water ‘ad libitum’ before and during the experiments, and they were kept in an environment with an average temperature of 22 ± 2 °C with a normal 12/12-h light/dark cycle. All efforts were made to minimize the number of animals used and reduce their suffering during the experiments.

Molecular analyses of the study were performed in Selcuk University Medical Faculty Molecular Physiology Laboratory, histological analyses were performed in the Selcuk University Medical Faculty Histology Laboratory.

### Bilateral Common Carotid Arteries Occlusion/Reperfusion (BCCAo/r) Model Establishment

Two-vessel occlusion model (2VO) was used to generate experimental global brain ischemia in rats. Surgical procedures performed with minor changes as reported in the previous study of the same authors (Caliskan et al., 2016). In brief, the animals were first anesthetized by intraperitoneal (i.p.) administration of ketamine HCl (60 mg/kg) and xylazine (5 mg/kg). Then anesthetized rats were placed in a supine position on the operating table, and a cervical incision was made in the ventral midline to expose the right and left common carotid arteries.

After the bilateral common carotid arteries were carefully isolated from surrounding tissues, ligation was performed for 30 min to induce global cerebral ischemia. After 30 min, the ligation was untied, and the incision was closed after the re-establishment of blood flow was confirmed visually. To determine the effect of anesthesia and surgical manipulation on the results, later the right and left common carotid arteries were carefully isolated in the sham animals, the neck incision was closed without occlusion.

### Animal Grouping and Drug Administration

**Fig. 1:**
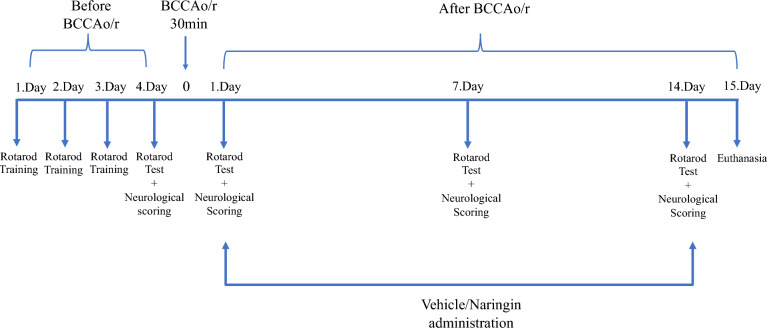
Timeline of the study. Plan of performing rotarod tests and neurological scoring before and after bilateral common carotid artery occlusion/reperfusion (BCCAo/r)

40 male Wistar rats were randomly divided into 5 groups as follows: (1) Control (*n* = 6), (2) Sham (*n* = 6), (3) Ischemia–Reperfusion (I/R) (*n* = 9), (4) Ischemia–Reperfusion + Vehicle (I/R + V) (*n* = 9), and (5) Ischemia–Reperfusion + Naringin (I/R + N) (*n* = 10). Animals in the I/R + V group received 0.25% sodium carboxymethyl-cellulose (CMC-Na) as a vehicle for 2 weeks. For animals in the I/R + N group, naringin was dissolved in 0.25% CMC-Na at a dose of 100 mg/kg and administered by intragastric route once daily for consecutive 14 days after BCCAo/r (Fig. 1).

### Neurological Evaluation

We evaluate the motor functions of the rats one day before BCCAo/r establishment, to determine the baseline before the insult, and at 1, 7, and 14 days after the insult to identify the success of the BCCAo after reperfusion and assess the severity of the injury and whether it changes with the naringin administration over 14 days. Rats were scored on a scale of 0–3 (normal score, 0; maximal deficit score, 3) according to the Bederson scoring system as previously described (Bederson et al., 1986).

The neurological evaluation system is as follows: 0: No functional defects, 1: Variable degrees of forepaw flexion, 2: Variable degrees of forepaw flexion with minimal or no resistance to lateral pressure, 3: Variable degrees of forepaw flexion, minimal, or no resistance to lateral pressure and circling behavior on walking.

### Rotarod Test

Motor function was assessed using an accelerating rotarod (Commat Ltd. TURKEY). All animals were given 3 consecutive days of 5-min training sessions and then tested on the fourth day before BCCAo/r to determine baseline performance. Rats were first accommodated to the apparatus by placing them on a steady rod. Then, the rod was started at 4 rpm and accelerated linearly to 40 rpm within 300 s. Rats that fell in the training sessions were positioned again on the rotating rod until the session was completed for 5 min. The average latency to fall off the rotating rod was recorded for each rat in seconds for three trials. The test was repeated after BCCAo/r at days 1, 7, and 14.

### Immunohistochemical Analysis

For DAPI and anti-NeuN markers, 40 hippocampus and 40 frontal cortex samples from rats were fixed with 4% paraformaldehyde. The desired cross-sectional surface was angled and carefully embedded into the cryomatrix. Serial sections of 5 μm thickness were taken on the cryostat device (Thermo Shandon Cryostat 210160 GB). Sections were treated first with PBS and then with Triton X100 for 10 min in a humidity box. After that, they were washed 3 times for 5 min each with PBS then incubated with the block solution for 30 min at room temperature and the remaining excess liquid was aspirated. For immunohistochemical labeling, samples were incubated with the primary antibody of rat anti-NeuN (ab279297) (1/100 diluted) for 24 h at 4 °C then incubated with the secondary antibody (DyLight 550 (ab96888) (1/100 diluted) for 2 h in the dark at room temperature. After the final wash with PBS, the sections were covered with a fluorescent occlusion medium with DAPI (Fluoroshield with DAPI, F6057, Sigma-Aldrich, USA). Examined under an Olympus BX51 Trinocular fluorescence microscope, the determined areas were recorded with a DP72 camera by randomly selecting 4 areas from each at 40X magnification. Field validation was done with DAPI nucleus dye. The ratio of anti-NeuN labeled cells to total cells in the recorded images was calculated using the Image J (National Institutes of Health, Bethesda, MD, USA) program.

### Real-Time Quantitative Polymerase Chain Reaction (RT-qPCR) Analysis

Total RNA from the hippocampus and frontal cortex tissues was first isolated using a commercial kit (Bio Basic; Canada) to determine the expression of DCX and BDNF genes by Real-Time qPCR analysis. The mRNA in total RNA obtained in the previous step was converted to cDNA using the commercial kit (OneScript Plus, ABM; Canada) according to the protocol. Nanodrop (SMA 1000, Merinton, China) was used to determine the amount and purity of cDNA obtained. Primers used for the analysis of target gene expression at the mRNA level were obtained from the manufacturer (Sentebiolab, Turkey) and presented in (Table 1). GAPDH synthesized by Oligomer (Turkey) company was used as a reference gene.

**Table 1:** Primers used for real-time quantitative polymerase chain reaction RT-qPCR

Gene	Primer sequence	Function
DCX forward	5′-TGGATGCATCAAAGGTGTGT-3′	Target Gene
DCX back	5′-TGCCAAACATACCAGTCCAA-3′	Target Gene
BDNF advanced	5′-GGGTGAAACAAAGTGGCTGT-3′	Target Gene
BDNF back	5′-ATGTTGTCAAACGGCACAAA-3′	Target Gene
GAPDH next	5′-GGGCCAAAAGGGTCATCATC-3′	Reference Gene
GAPDH back	5′-AACCTGGTCCTCAGTGTAGC-3′	Reference Gene

A ready commercial kit (BlasTaq 2X qPCR Master Mix, ABM, Canada) was used for PCR reactions. All reactions were performed using the CFX Connect Real-Time PCR Detection System (Bio-Rad, USA). qPCR analysis results were evaluated using the 2^−∆∆CT^ method developed by Livak and Schmittgen (Livak & Schmittgen, 2001).

### Statistical Analysis

The conformity of the quantitative data in the groups to the normal distribution was examined with the single-sample Kolmogorov–Smirnov test. In addition, a one-way analysis of variance was used to compare the quantitative values for each group in different time sections. *P* < 0.05 value was accepted as the statistical significance limit in the study.

## Results

### Naringin Improved Neurological Function After Ischemia/Reperfusion

**Fig. 2:**
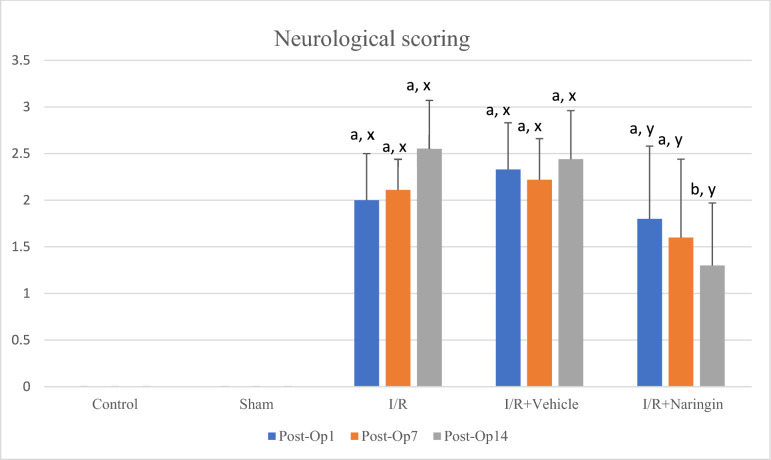
*a,*b*,*c* shows the statistical difference within the same group (*a* > *b* > *c*) (*P* < 0.001); *x*,*y*,*z* shows the statistical difference in groups (*x* > *y* > *z*) (*P* < 0.001). I/R led to increasing neurologic score levels, however, naringin supplementation improved neurologic score levels especially on days 7th and 14th after BCCAo/r

The intragroup and intergroup variations in neurological recovery among animals are shown in Table 2. When the intragroup evaluation was made, it was determined that group 3, 4, and 5 post-op values showed a significant increase compared to pre-op values. When the intergroup evaluation was made, it was seen that there was no difference in the pre-op evaluation, but there was a significant increase in post-op values in the I/R groups compared to the control and sham groups. However, neurologic score values showed a significant improvement after naringin supplementation (*P* < 0.001) (Fig. 2, Table 2). Considering the neurological scoring results, the scoring of the control and sham groups did not change on the 1st, 7th, and 14th days of pre-op and post-op. However, while neurologic scores increased in the I/R groups, naringin supplementation prevented this increase.

**Table 2:** Intragroup and intergroup neurologic scoring results

Groups	Pre-op	Post-op1	Post-op7	Post-op14
	(Mean ± SD)	(Mean ± SD)	(Mean ± SD)	(Mean ± SD)
Control	0,00 ± 0,00 c, z	0,00 ± 0,00 c, z	0,00 ± 0,00 c, z	0,00 ± 0,00 c, z
Sham	0,00 ± 0,00 c, z	0,00 ± 0,00 c, z	0,00 ± 0,00 c, z	0,00 ± 0,00 c, z
I/R	0,00 ± 0,00 c, z	2,00 ± 0,50 a, x	2,11 ± 0,33 a, x	2,55 ± 0,52 a, x
I/R + Vehicle	0,00 ± 0,00 c, z	2,33 ± 0,50 a, x	2,22 ± 0,44 a, x	2,44 ± 0,52 a, x
I/R + Naringin	0,00 ± 0,00 c, z	1,80 ± 0,78 a, y	1,60 ± 0,84 a, y	1,10 ± 0,31 b, y

*a,*b*,*c* shows the statistical difference in the same row (*a* > *b* > *c*) (*P* < 0.001); *x*,*y*,*z* shows the statistical difference in the same column (*x* > *y* > *z*) (*P* < 0.001). I/R led to increasing neurologic score levels, however, naringin supplementation improved neurologic score levels especially on days 7th and 14th after BCCAo/r

### Naringin Improved Motor Function After Ischemia/Reperfusion

**Fig. 3:**
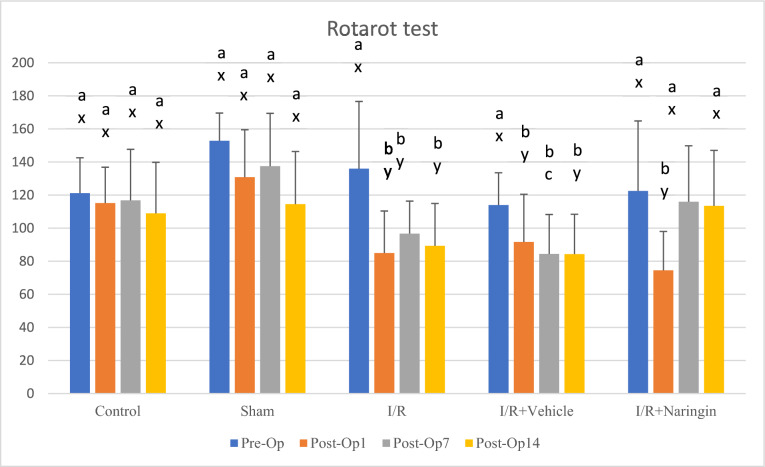
*a,*b*,*c* shows the statistical difference within the same group; *x*,*y*,*z* shows the statistical difference in the groups (*a* > *b* > *c*; *x* > *y* >) (*P* < 0.001). I/R led to increasing rotarod test values, however, naringin supplementation increased rotarod test values back to control levels on the 7th and 14th days after BCCAo/r

Rotarod test results are shown in Table 3. In the comparison of the groups, it was determined that I/R significantly reduced latency to fall values in groups 3, 4, and 5. However, in Group 5, 2 weeks of naringin supplementation increased the latency to fall values that reduced due to I/R back to control values on days 7th and 14th after BCCAo/r (*P* < 0.001) (Table 3, Fig. 3).

**Table 3:** Intragroup and intergroup rotarod test results

Groups	Pre-op	Post-op1	Post-op7	Post-op14
	(Mean ± SD)	(Mean ± SD)	(Mean ± SD)	(Mean ± SD)
Control	121,17 ± 21,40 a, x	115,17 ± 21,69 a, x	116,83 ± 30,84 a, x	108,97 ± 30,83 a, x
Sham	152,83 ± 16,76 a, x	130,83 ± 28,68 a, x	137,50 ± 31,91 a, x	114,50 ± 31,88 a, x
I/R	136,00 ± 40,62 a, x	85,00 ± 25,38 b, y	96,67 ± 19,68 b, y	89,33 ± 25,60 b, y
I/R + Vehicle	114,00 ± 19,48 a, x	91,67 ± 28,84 b, y	84,44 ± 23,83 bc, y	84,33 ± 24,08 b, y
I/R + Naringin	122,50 ± 42,33 a, x	74,50 ± 23,51 b, y	116,00 ± 33,81 a, x	113,50 ± 33,54 a, x

*a,*b*,*c* shows the statistical difference in the same row, *x*,*y*,*z* shows the statistical difference in the same column (*a* > *b* > *c*; *x* > *y* >) (*P* < 0.001). I/R led to increasing rotarod test values, however, naringin supplementation increased rotarod test values back to control levels on the 7th and 14th days after BCCAo/r

### Naringin Improved Neurogenesis After Ischemia/Reperfusion

**Fig. 4:**
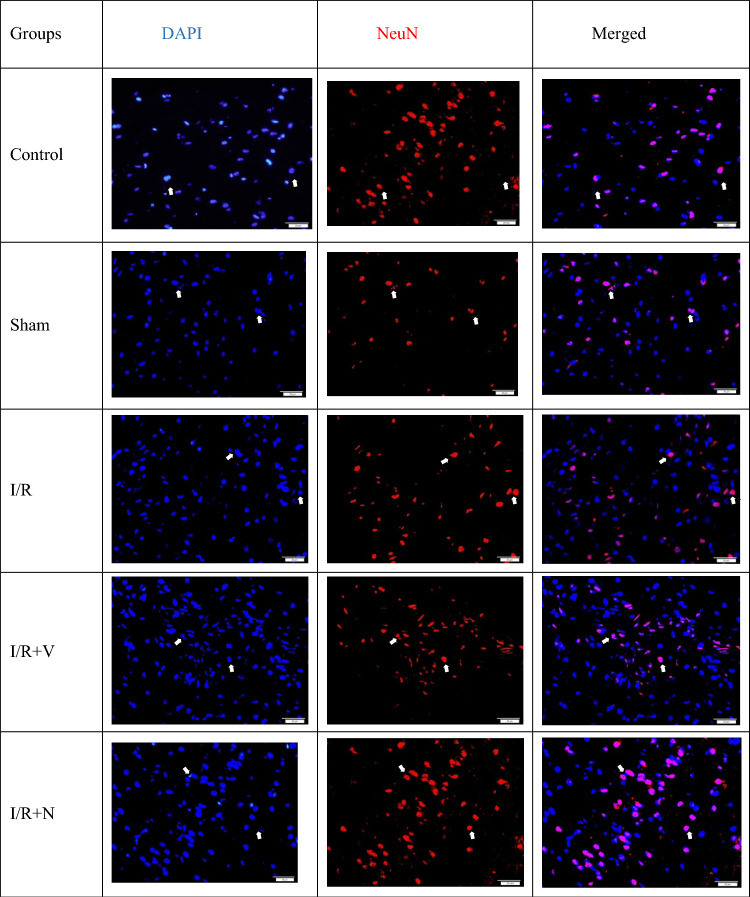
Fluorescent images with immunolabeling to demonstrate neurogenesis in the frontal cortex. DAPI (blue) column shows all nucleated cells (neurons and glia) in frontal cortex tissue, NeuN (red) column shows mature neurons in frontal cortex tissue, DAPI and NeuN images combined (pink) are shown in merged column. Arrows indicate mature neurons as examples (X40 Magnification; 1 bar: 20 µm)

**Fig. 5:**
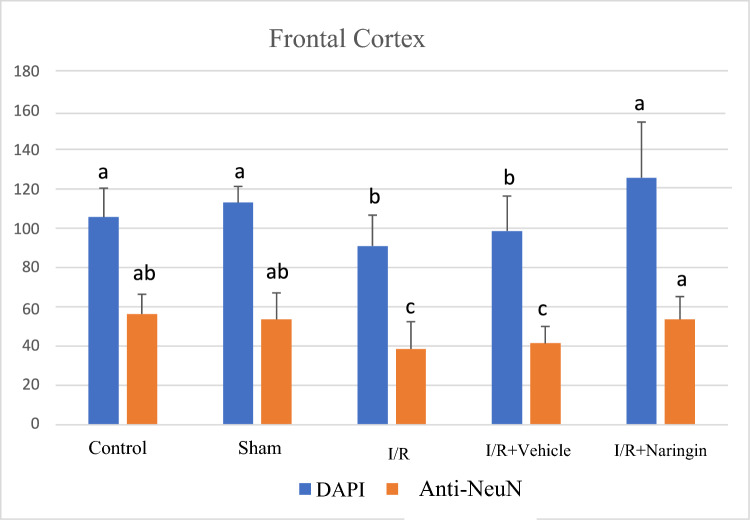
Levels of DAPI and anti-NeuN antibodies in the frontal cortex in experimental groups. While the number of cells stained with DAPI and NeuN was unchanged in the control and sham groups, it decreased in the I/R and I/R + solvent groups, but increased significantly with naringin supplementation (*a* > *b* > *c*; *P* < 0.001)

**Fig. 6:**
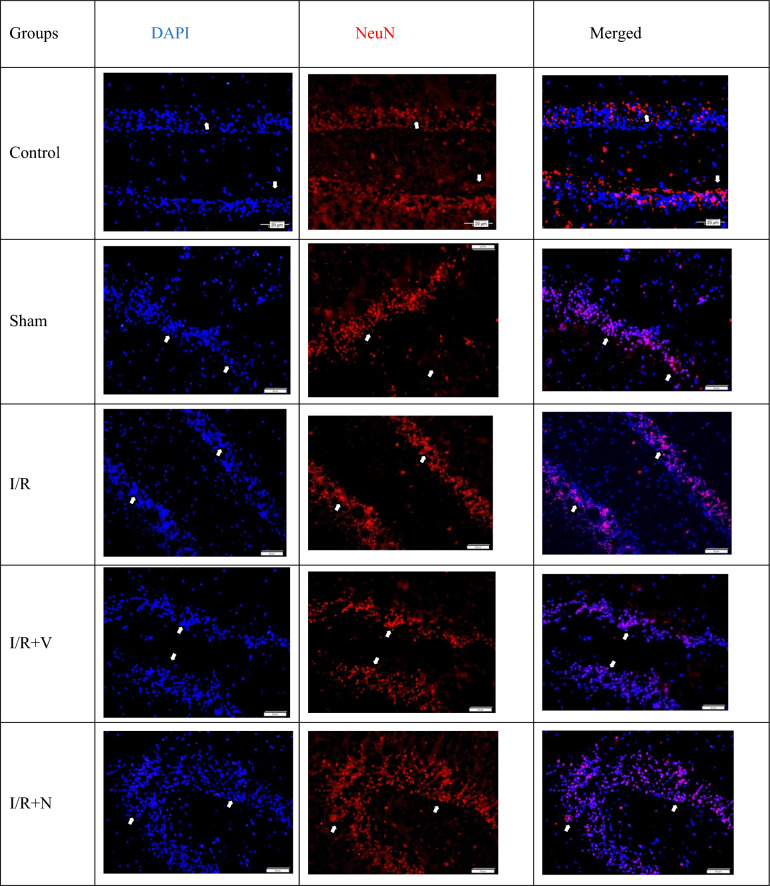
Fluorescent images with immunolabeling to demonstrate neurogenesis in the hippocampus. The DAPI (blue) column shows all nucleated cells (neurons and glia) in the hippocampus tissue, the NeuN (red) column shows mature neurons in the hippocampus tissue, the merged (pink) version of the DAPI and NeuN images is shown in the merged column. Arrows indicate mature neurons as examples (X40 Magnification; 1 bar: 50 µm)

**Fig. 7:**
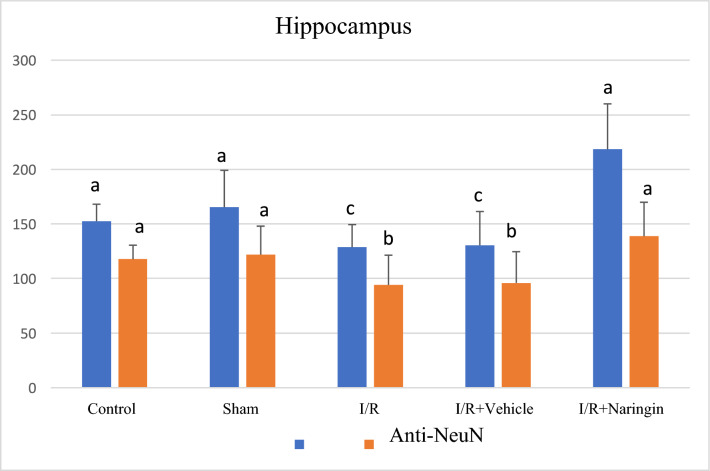
Levels of DAPI and anti-NeuN antibodies in the hippocampus in experimental groups. While the number of cells stained with DAPI and NeuN did not change in the control and sham groups, it decreased in the I/R and I/R + solvent groups, but increased significantly with naringin supplementation (*a* > *b* > *c*; *P* < 0.001)

The expression levels of the DAPI (marker expressed by all nucleated cells) and the anti-NeuN antibody (expressed by mature neurons) in the frontal cortex and hippocampus tissues are shown in Tables 4 and 5; Figs. 4, 5, 6, and 7.

**Table 4:** The expression level of DAPI and NeuN markers in the frontal cortex

Groups	DAPI (Mean ± SD)	NeuN (Mean ± SD)
Control	105,53 ± 14,75 a	56,31 ± 10,06 ab
Sham	112,92 ± 8,23 a	53,60 ± 13,46 ab
I/R	90,90 ± 15,65 b	38,54 ± 13,87 c
I/R + Vehicle	98,56 ± 17,69 b	41,41 ± 8,59 c
I/R + Naringin	125,66 ± 28,25 a	53,57 ± 11,62 a

Different letters in the same column are statistically significant (*a* > *b* > *c*; *P* < 0.001). I/R led to reducing DAPI and NeuN marker levels in groups 3 and 4 on the 15th day after BCCAo/r, but naringin supplementation has enhanced the deteriorated levels of DAPI and NeuN back to control levels

**Table 5:** The expression level of DAPI and NeuN marker in the hippocampus

Groups	DAPI (Mean ± SD)	NeuU (Mean ± SD)
Control	152,46 ± 15,59 ab	117,71 ± 12,82 a
Sham	165,25 ± 33,86 ab	121,96 ± 26,03 a
I/R	128,62 ± 20,79 c	94,18 ± 27,19 b
I/R + Vehicle	130,41 ± 30,93c	95,93 ± 28,70 b
I/R + Naringin	218,30 ± 41,74 a	138,73 ± 31,16 a

Different letters in the same column are statistically significant (*a* > *b* > *c*; *P* < 0.001). I/R led to reduced levels of DAPI and NeuN markers in groups 3 and 4 on the 15th day after BCCAo/r, but these levels have improved back to control levels with naringin supplementation in group 5

According to the data obtained, cell proliferation in the frontal cortex (indicated by the DAPI marker) and mature neuron numbers (indicated by the NeuN marker) were at a similar level in groups 1 and 2, and the level of these parameters decreased in groups 3 and 4. In the Naringin-supplemented group, cell proliferation and neuronal maturation showed a significant enhancement represented by increased DAPI and NeuN marker levels to control levels.

Based on the data obtained from the hippocampus, the DAPI and NeuN marker levels were the same as the results in the frontal cortex. The DAPI and NeuN markers were at close levels in the control and sham groups. 14 days after BCCAo/r, DAPI and NeuN marker levels were low in I/R and I/R + Vehicle groups. However, naringin supplementation for 2 weeks restored low values to control levels.

**Fig. 8:**
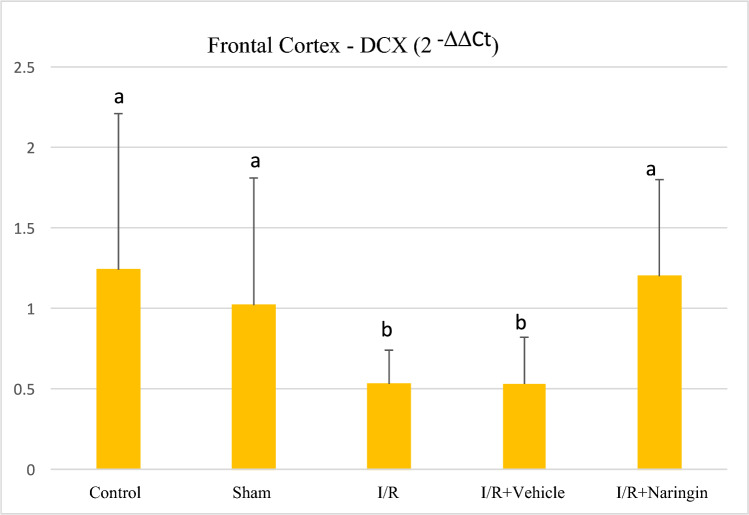
Expression level of DCX gene in the frontal cortex in experimental groups. Expression levels of the DCX gene did not change in the control and sham groups, but decreased in the I/R and I/R + solvent groups. With naringin supplementation, it again reached control group levels (*a* > *b*; *P* < 0.03)

**Fig. 9:**
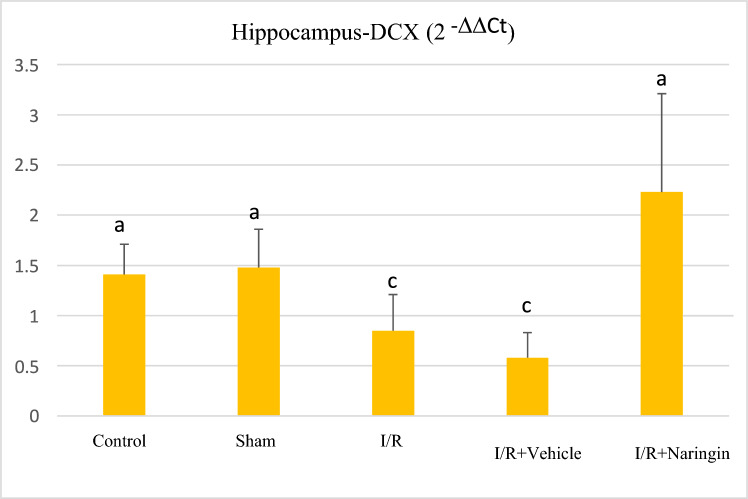
Expression level of DCX gene in the hippocampus in experimental groups. Expression levels of the DCX gene did not change in the control and sham groups, but decreased in the I/R and I/R + solvent groups. With naringin supplementation, it increased back to the control group levels (*a* > *b* > *c*; *P* < 0.001)

The expression level of DCX gene (gene expressed by newly born neurons) assessed by RT-qPC in the frontal cortex and hippocampus tissues is shown in Table 6; Figs. 8 and 9.

**Table 6:** The expression level of the DCX gene in the frontal cortex and Hippocampus

Groups	Frontal Cortex	Hippocampus
	(2 ^−ΔΔCt^ Mean ± SD)	(2^−ΔΔCt^ Mean ± SS)
Control	1,24 ± 0,97 a	1,41 ± 0,30 ab
Sham	1,02 ± 0,79 a	1,48 ± 0,38 ab
I/R	0,53 ± 0,21 b	0,85 ± 0,36 c
I/R + Vehicle	0,53 ± 0,29 b	0,58 ± 0,25 c
I/R + Naringin	1,20 ± 0,60 a	2,23 ± 0,98 a

Different letters in the same column are statistically significant (*a* > *b* > *c*; frontal cortex: *P* < 0.03; Hippocampus: *P* < 0.001). DCX gene expression in the frontal cortex and hippocampus was at the same level in the control and sham groups. In I/R and I/R + Vehicle groups, the DCX gene expression level decreased on the 15th day after BCCAo/r but with naringin supplementation, the DCX gene expression level rose back to control levels

Based on the data obtained from both the frontal cortex and hippocampus, there was no statistical variation in DCX gene expression between the control and sham groups. The DCX gene expression level was low in ischemia-induced groups (I/R and I/R + Vehicle) and this deterioration was recovered with naringin supplementation (group 5) and increased to the control group level.

### Naringin Improved BDNF Levels After Ischemia/Reperfusion

**Fig. 10:**
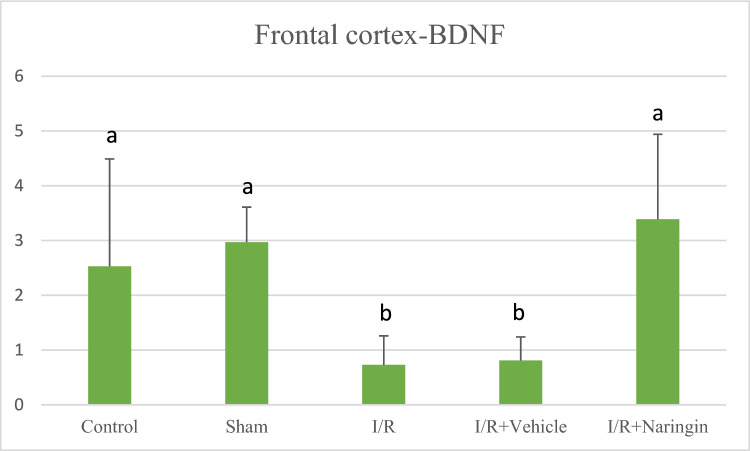
Expression level of BDNF gene in the frontal cortex in experimental groups. While the BDNF gene expression level was close in the control and sham groups, it decreased in the I/R and I/R + solvent groups, but this decrease was restored with naringin supplementation and increased to the control level (*a* > *b*; *P* < 0.01)

**Fig. 11:**
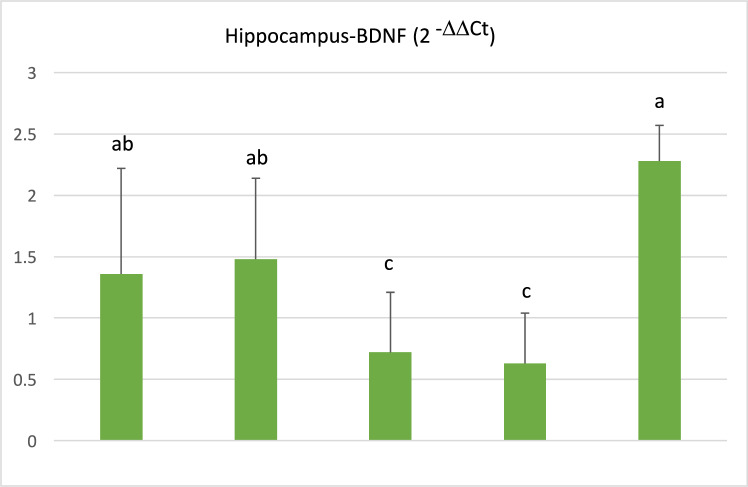
Expression level of BDNF gene in the hippocampus in experimental groups. BDNF gene expression levels were close in the control and sham groups. The decreased BDNF gene expression in the I/R and I/R + solvent groups reached control level again with naringin supplementation (*a* > *b* > *c*; *P* < 0.01)

The expression level of the BDNF gene in the frontal cortex and hippocampus tissues is presented in (Table 7; Figs. 10, 11). According to the data provided above regarding BDNF gene expression in both the frontal cortex and hippocampus tissues, no difference could be detected between control and sham groups, while in I/R and I/R + Vehicle groups it reduced on 15th day after BCCAo/r. However, with naringin supplementation, BDNF gene expression increased back to control levels.

**Table 7:** The expression level of BDNF gene in the frontal cortex

Groups	Frontal Cortex	Hippocampus
	(2 ^−ΔΔCt^ Mean ± SD)	(2 ^−ΔΔCt^ Mean ± SD)
Control	2,53 ± 1,96 a	1,36 ± 0,86 ab
Sham	2,97 ± 0,64 a	1,48 ± 0,66 ab
I/R	0,73 ± 0,53 b	0,72 ± 0,49 c
I/R + Vehicle	0,81 ± 0,43 b	0,63 ± 0,41 c
I/R + Naringin	3,39 ± 1,55 a	2,28 ± 0,29 a

Different letters in the same column are statistically significant (*a* > *b* > *c*; frontal cortex: *P* < 0.01; hippocampus: *P* < 0.01). BDNF gene expression level did not show any difference between the control and sham groups in both the frontal cortex and hippocampus. Ischemia induction resulted in BDNF gene expression level reduction in I/R and I/R + Vehicle groups then this reduction was restored to control levels with 14 days-naringin supplementation in the I/R + Naringin group

## Discussion

### Effect of Naringin on Neurological and Motor Functions After Ischemia/Reperfusion

When we look at the findings of the study in general, it is seen that ischemia–reperfusion in rats caused functional damage as expected. This functional damage was observed in both neurological scoring (increased score values) and rotarod test (decreased latency time of falling). Another important result of this study is that ischemia–reperfusion caused a significant decrease in BDNF levels as well as neurogenesis, depending on the ischemia–reperfusion time. On the other hand, a 2-week naringin supplementation restored the deteriorated neurogenesis and BDNF levels due to ischemia–reperfusion.

When the neurological score values of the experimental groups were examined, ischemia–reperfusion significantly increased these values, while naringin supplementation significantly suppressed them. Many flavonoids have been used in numerous studies to demonstrate their effect on neuronal and functional recovery after stroke. Flavonoids exert their Neuroprotective effects via interaction with various intracellular pathways essential for controlling neuronal survival, differentiation, migration, regeneration, and neurogenesis (Spencer et al., 2009). ( −)-Epigallocatechin-3‐gallate (EGCG), a flavonoid found in abundant in green tea, was administered to mice subjected to 60-min focal cerebral ischemia by intracerebroventricular injection for 2 weeks starting from day 14 after reperfusion. Twenty-eight days after ischemia, EGCG treatment increased cell proliferation and migration to the ischemic striatum, and functional improvement was observed (Zhang et al., 2017).

Administration of naringin at different doses to experimental animal models of ischemia–reperfusion induced for variable durations of time has demonstrated a positive effect on neurological function. Naringin (80, 120, or 160 mg/kg) has been reported to reduce neurologic deficit scoring when given as a single dose via the femoral vein to rats subjected to a 2-h focal cerebral ischemia (Feng et al., 2018). In another study, intragastric administration of naringin (25, 50, and 100 mg/kg) for 7 days before exposure to 2-h global cerebral ischemia improved neurologic function in rats after ischemia–reperfusion injury (Wang et al., 2021). Moreover, pre-treatment of naringin (5 mg/Kg) for 7 days reduced neurologic defect scores and apoptosis of neurons in the hippocampus in rats subjected to middle cerebral artery occlusion (Yang et al., 2020). In rats who received 7-day treatment of naringin (50–100 mg/kg) prior to 30-min bilateral common carotid arteries occlusion, the neurological function significantly improved after 24 h of reperfusion (Gaur et al., 2009). In our study, the fact that intragastric naringin treatment (100 mg/kg) for 14 days after ischemia–reperfusion showed a significant improvement in neurological scores is similar to the studies mentioned above.

To evaluate the motor coordination and balance of experimental animals, we performed the rotarod test which was first described by Dunham and Miya for assessing motor coordination in rodents (Dunham & Miya, 1957) by recording the time spent by the animals on the rotating rod (latency to fall) (Lubrich et al., 2022). According to the test, in I/R and I/R + Vehicle groups, latency to fall decreased significantly, while 2 weeks of naringin treatment brought the results closer to the control and sham groups’ results. Mice that experienced experimental stroke by occluding the left middle cerebral artery showed a reduced latency to fall compared to control and sham-operated mice on day 1 after ischemia indicating that ischemia had induced a marked deficit in motor coordination and balance. However, there was no significant difference between the two groups on day 7 due to an increase in time spent on the rotarod by the ischemic group, demonstrating recovery of performance on this task with time (Hunter et al., 2000). In another study, rats with ischemic lesions were subjected to the rotarod test on the 7th, 14th, 21st, and 28th days after ischemia. At postoperative day 7, the ischemic groups showed significantly lower values than the sham group. After the 14th postoperative day, the values in the ischemia group remained significantly lower than the values in the sham group until the postoperative 28th day, and the ischemic group did not show any improvement in motor dysfunction (Tamakoshi et al., 2023).

In another experimental study, it was found that the enriched environment supported the recovery of limb function in mice subjected to focal cerebral ischemia, as assessed by the modified neurological severity score and the rotarod test at 3, 7, 14, and 21 days after ischemia (Shi et al., 2023). In the studies where brain ischemia–reperfusion was performed, it was seen that the improvement in limb function led to the emergence of different responses depending on the experimental model difference. The decrease in latency to fall in the rotarod test, which was induced by occluding the two common carotid arteries for 30 min and continued for 2 weeks after reperfusion, was restored to pre-ischemic levels in the naringin-treated group, especially on the post-op 7th and 14th days. This result shows the effectiveness of naringin treatment at the dose and duration applied in our experiment.

### Effect of Naringin on Neurogenesis After Ischemia/Reperfusion

Other parameters we examined in our study were DAPI, NeuN, and DCX as indicators of neurogenesis. While all these mentioned parameters were significantly suppressed with ischemia–reperfusion, intragastric naringin treatment at a dose of 100 mg/kg for 2 weeks increased the reduced values back to their normal levels and even above them.

Altman and Das (Altman & Das, 1965) were the first to identify adult neurogenesis in the dentate gyrus of the hippocampus since then, many studies have been conducted to detect neurogenesis in other brain regions. Later, it was determined that focal ischemia can trigger neurogenesis in the usual neurogenic niches, i.e., subventricular zone (SVZ) of the lateral ventricle and the subgranular zone (SGZ) of the dentate gyrus (Jhelum et al., 2022; Wiltrout et al., 2007). Now it is believed that many regions of our brain (eg, hypothalamus, neocortex, striatum, amygdala, etc.) are capable of neurogenesis and maintains this ability throughout adult life (Bellenchi et al., 2013; Gu et al., 2000; Kuhn et al., 2018). And also Kreuzberg et al. (Kreuzberg et al., 2010) have reported that Post-stroke-generated SVZ-derived neuroblasts migrated to the cortex and survived for at least 35 days representing 2% of BrdU-positive cells in peri-infarct area, where they differentiate into mature neurons. Thus, stroke enhances SVZ neurogenesis and attracts newborn neurons to the injury zone. As stated in the above publications, since the migration of newborn neurons to different brain regions and maturation in those regions occur during the neurogenesis process, we aimed to determine the effect of 2-week naringin treatment after focal brain ischemia–reperfusion on neurogenesis in the hippocampus and frontal cortex.

DCX is widely used as an indicator of neurogenesis and a marker for labeling newly born neurons (Bregere et al., 2017; Couillard-Despres et al., 2005). DCX has been detected in the axons and dendrites of newly born granule cells in the early stages of cell proliferation up to the postmitotic phase and has been accepted as a marker of immature granule cells since it disappears from mature granule cells (Plumpe et al., 2006). NeuN is a nuclear protein that is expressed almost exclusively in mature neurons of the peripheral and central nervous system (Mullen et al., 1992). NeuN is distributed in the nuclei of mature neurons in nearly all parts of the vertebrate nervous system, therefore, it has been accepted as a reliable marker of mature neurons (Duan et al., 2016). The effects of different flavonoids on neurogenesis have been investigated in different studies that have been carried out before. Indeed, rats subjected to a 60-min focal cerebral ischemia, treated with EGCG (2.5 mg/kg) for 14 days resulted in a high number of newly born DCX-labeled cells in the hippocampus (Ortiz-Lopez et al., 2016). The effect of 7,8-dihydroxyflavone (7,8-DHF), a flavone derivative, was studied both in vivo and in vitro in a mouse model of Down syndrome. In vitro, neural stem/progenitor cell culture from SVZ treated with 7,8-DHF showed no increase in proliferation, but increased differentiation of neural stem/progenitor cells into MAP2 + mature neurons. It has been shown that 7,8-DHF (5 mg/kg) when administered on postnatal days 3–15 greatly increased the number of BrdU + newly born cells in the dentate gyrus, but the number of proliferating cells did not reach the same value as in euploid mice (Stagni et al., 2017). The effect of Chrysin, another flavonoid, on hippocampal neurogenesis and memory was investigated when administered at doses of 10 or 30 mg/kg/day for 8 weeks after induction of brain aging by D-galactose administration in rats. Chrysin treatment increased the number of Ki67 + cells (a marker for proliferated cells) in the SGZ, improved cell survival, and preserved the number of immature DCX + neurons (Prajit et al., 2020). When the effect of citrus flavonoid 3,5,6,7,8,31,41-heptamethoxyflavone (HMF) on neurogenesis is examined in the depression model in SGZ, HMF restored the reduction of neurogenesis levels indicated by increased DCX + -labeled cells in hippocampal SGZ at day 26 of treatment in these animals (Sawamoto et al., 2016).

Many flavonoids have been used in numerous studies to demonstrate their effect on neuronal and functional recovery after ischemia–reperfusion. When EGCG was administered by intracerebroventricular injection for 2 weeks starting from day 14 after reperfusion to mice subjected to 60-min of focal cerebral ischemia, at 28 days post ischemia, EGCG treatment enhanced cell proliferation (indicated by BrdU, Ki67, and BrdU/DCX-labeled cells) and migration (indicated by the BrdU/DCX-labeled cells) in ischemic striatum (Zhang et al., 2017). HMF also increased the number of immature DCX + neurons in both the SGZ and SVZ when HMF (low dose 25 or high-dose 50 mg/kg/day) was maintained in mice 5 days before and 3 days after induction of 12-min global cerebral ischemia (Okuyama et al., 2012). In another study, after inducing 12-min transient global ischemia in mice, HMF administered for 3 days increased neurogenesis as assessed by high DCX + cell count in SGZ (Okuyama et al., 2014). When Baicalin was administered to rats at a dose of 50 mg/kg once daily for 3 weeks after 6 min of transient forebrain ischemia, it stimulated the number of newly born BrdU + cells and their differentiation into mature BrdU/NeuN + neurons in the dentate gyrus SGZ, associated with facilitated performance in the maze test indicating cognitive improvement (Zhuang et al., 2013). ASF, a flavonoid, was administered to rats exposed to focal cerebral ischemia at doses of 2.5, 5, and 7.5 mg/kg for 7 and 14 days. ASF treatment improved neurological functions and increased the proliferation of neural stem/progenitor cells after cerebral ischemia (Gao et al., 2022b). In Medline searches, only one study was found determining the effect of naringin treatment on neurogenesis (Yang et al., 2018). We also looked at IL-10 levels as part of the research to determine the inflammatory process, but the data should not be used here (unpublished information). However, in studies conducted with the use of different flavonoids other than naringin, it has been reported that neurogenesis increases by suppressing neuroinflammation (Rostamian et al., 2023; Tang et al., 2023).

In some previous studies, the effect of naringin on new neuron development was investigated. In one study, it was determined that under normal physiological conditions, when naringin was given to mice at a dose of 5 mg/kg for 5 days, it increased the differentiation of cells to mature neurons in the hippocampus. In SVZ, naringin treatment promoted neuronal differentiation (increased BrdU + /NeuN + cell count). In the second experiment of the same study, when 50 mg/kg/day naringin was administered for 25 days, starting from the 15th day after the initiation of corticosterone treatment to produce depressive-like effects in mice, it was found that BrdU + /DCX + newly born neurons in the dentate gyrus increased and promoted their development into mature neurons represented by high numbers of BrdU + / NeuN + cells (Gao et al., 2022a). It was found that when naringin (100 mg/kg) was administered to a mouse model of hyperglycemia diabetes along with auraptene for 14 days, it increased the number of DCX + immature neurons in the SGZ of the hippocampus (Okuyama et al., 2018). In our study, intragastric naringin supplementation at a dose of 100 mg/kg for 2 weeks after 30-min global cerebral ischemia induction markedly increased neurogenesis by restoring the ischemia/reperfusion-induced suppression of both the immature neuron marker (DCX) and the mature neuron marker (NeuN).

### Effect of Naringin on BDNF Levels After Ischemia/Reperfusion

The experimental study we conducted examined the effect of I/R and naringin supplementation on BDNF levels. While this parameter was significantly reduced by I/R, 2 weeks of naringin supplementation significantly increased BDNF levels.

BDNF is a member of the neurotrophin family, and among all neurotrophins, BDNF is the best known and studied in the central nervous system, given the fact that it regulates many different cellular processes involved in the development and maintenance of normal brain function (Leal-Galicia et al., 2021). BDNF modulates adult neurogenesis by regulating neuronal differentiation and survival (Bath et al., 2012; Lee et al., 2002).

In a conducted study, it was found that BDNF overexpression in the hippocampus increased the proliferation and survival of newly born granule cells and maturation, represented by DCX + cells with tertiary dendrites (Quesseveur et al., 2013). Exogenous administration of BDNF has also been used to shed light on the mechanisms underlying the effects of this neurotrophin on adult neurogenesis. Delivery of BDNF to the hippocampus by bionanoparticles increased proliferation and survival of newly born granule cells, synaptogenesis, and dendritic morphogenesis in the hippocampus in an iTat mouse model of the HIV/neuroAIDS (Vitaliano et al., 2022). Increased neurogenesis in the dentate gyrus was observed after BDNF infusion into the hippocampus of adult rats (Scharfman et al., 2005).

The link between BDNF and the modulation of neurogenesis by external stimuli has been a topic that has been extensively studied in recent years. Many chemicals have been proven to upregulate neurogenesis by modulating BDNF expression (Numakawa et al., 2017). Heterozygous BDNF gene knockout (BDNF ±) in mice resulted in down-regulation of BDNF in the hippocampus and thus neural cell proliferation and survival but was up-regulated after dietary restriction to a lesser extent (Lee et al., 2002). Many studies have shown that BDNF may have a regulatory effect on adult neurogenesis after neurological events such as subarachnoid hemorrhage and ischemia (Leal-Galicia et al., 2021). There are numerous studies investigating the effect of BDNF as a therapeutic agent administered directly to the brain or intravenously for the treatment of stroke, due to its role in infarct size reduction, attenuation of neuronal apoptosis, enhancement of neurogenesis/migration, and post-infarction functional outcome (Liu et al., 2020). BDNF loaded into exosomes injected into the striatum of the ischemic hemispheres 3 days after the induction of focal cerebral ischemia stimulated the differentiation of proliferating cells into neurons and consequently improved neurological functions (Zhu et al., 2023). Intravenous administration of BDNF for 5 days post stroke increased hippocampal newly born BrdU + cell count, mature BrdU/NeuN + neuron count, and neural stem/progenitor cell migration from the SVZ to the ischemic region in the striatum, up to 5 weeks after the last injection (Schabitz et al., 2007).

Modulation of BDNF levels, with factors such as enriched environment, increased BDNF level and thus SVZ neurogenesis. In contrast, the down-regulation of BDNF, for example by silencing Gadd45b gene expression, resulted in decreased SVZ proliferation and differentiation at the infarct border following ischemic injury (Tan et al., 2021). Naringin has promoted neurogenesis in the hippocampus via activation of BDNF/TrkB/CREB signaling pathway, both under physiological conditions when given at a dose of 5 mg/kg/day for 5 days and when administered to an animal model evoked depression at a dose of 10 or 50 mg/kg/day for 25 days (Gao et al., 2022a). In a rat model with diabetes, when naringin supplementation was administered at doses of 10, 20, and 40 mg/kg/day for 4 weeks, it was observed that BDNF levels increased and reached the highest level at a dose of 40 mg/kg/day (Ahmad et al., 2022). Naringin (20 mg/kg) improved the abnormal expressions of the PKA/CREB/BDNF pathway in the hippocampus 2 h after a single administration in chronically depressed mouse models (Wang et al., 2023). When naringin 100 mg/kg/day was administered orally to Alzheimer's model rats for 20 days, it was observed that it increased BDNF levels and improved memory in the new object recognition test (Choi et al., 2023). In our study, consistent with the results of the studies mentioned above, when naringin 100 mg/kg/day was administered intragastrically to rats for 14 days, it was observed that it increased the suppressed BDNF levels after ischemia/reperfusion injury.

## Conclusıon and Recommendatıons

The findings of the study show that 30 min of global brain ischemia followed by 2 weeks of reperfusion causes neurologic and motor deficits in rats. It has also been demonstrated that the same experimental model significantly suppresses neurogenesis. In this suppression, newly born immature neuron cells (DCX) and mature neuron cells (NeuN) were important mediator molecules. While BDNF levels, another important factor related to neurogenesis, decreased with ischemia–reperfusion, intragastric naringin treatment for 2 weeks significantly improved the BDNF levels.

In future studies, revealing the changes of different neurogenesis markers and important molecules in signaling pathways such as TrkB, MAPK/ERK, PI3K, and PLC-γ will provide a more detailed understanding of the mechanisms at the molecular level.

## Data Availability

The datasets which were generated during the current study are available from the corresponding author on reasonable request.

## Abbreviations

2VO: Two**-**vessel occlusion model
7,8-DHF: 7,8-dihydroxyflavone
BCCAo/r: Bilateral Common Carotid Arteries Occlusion/Reperfusion
BDNF: brain-derived neurotrophic factor
CMC-Na: sodium carboxymethyl-cellulose
CREB: cAMP response element-binding protein
DCX: Double cortin marker
EGCG: ( −)-Epigallocatechin-3‐gallate
ERK: Extracellular signal-regulated kinases
HMF: 3,5,6,7,8,31,41-heptamethoxyflavone
i.p.: intraperitoneal
MAPK: Mitogen-activated protein kinase
NeuN: neuronal nuclear antigen marker
PI3K: Phosphatidylinositol 3-kinase
PKA: protein kinase A
PLC-γ: Phospholipase C Gamma
RT-qPCR: Real-Time Quantitative Polymerase Chain Reaction
SGZ: subgranular zone
SVZ: subventricular zone
tPA: tissue plasminogen activator

## References

[CR1] Ojaghihaghighi, S., Vahdati, S. S., Mikaeilpour, A., & Ramouz, A. (2017). Comparison of neurological clinical manifestation in patients with hemorrhagic and ischemic stroke. *World Journal of Emergency Medicine,**8*, 34–38. 28123618 10.5847/wjem.j.1920-8642.2017.01.006 PMC5263033

[CR2] Prabhakaran, S., Ruff, I., & Bernstein, R. A. (2015). Acute stroke intervention: A systematic review. *JAMA,**313*, 1451–1462. 25871671 10.1001/jama.2015.3058

[CR3] Dorado, L., Millan, M., & Davalos, A. (2014). Reperfusion therapies for acute ischemic stroke: An update. *Current Cardiology Reviews,**10*, 327–335. 24646159 10.2174/1573403X10666140320144637 PMC4101197

[CR4] Rahman, A. A., Amruta, N., Pinteaux, E., & Bix, G. J. (2021). Neurogenesis after stroke: A therapeutic perspective. *Translational Stroke Research,**12*, 1–14. 32862401 10.1007/s12975-020-00841-w PMC7803692

[CR5] Marques, B. L., Carvalho, G. A., Freitas, E. M. M., Chiareli, R. A., Barbosa, T. G., Di Araujo, A. G. P., Nogueira, Y. L., Ribeiro, R. I., Parreira, R. C., Vieira, M. S., Resende, R. R., Gomez, R. S., Oliveira-Lima, O. C., & Pinto, M. C. X. (2019). The role of neurogenesis in neurorepair after ischemic stroke. *Seminars in Cell & Developmental Biology,**95*, 98–110. 30550812 10.1016/j.semcdb.2018.12.003

[CR6] Bartkowska, K., Tepper, B., Turlejski, K., & Djavadian, R. (2022). Postnatal and adult neurogenesis in mammals, including marsupials. *Cells,**11*(17), 2735. 36078144 10.3390/cells11172735 PMC9455070

[CR7] Culig, L., Chu, X., & Bohr, V. A. (2022). Neurogenesis in aging and age-related neurodegenerative diseases. *Ageing Research Reviews,**78*, 101636. 35490966 10.1016/j.arr.2022.101636 PMC9168971

[CR8] Accogli, A., Addour-Boudrahem, N., & Srour, M. (2020). Neurogenesis, neuronal migration, and axon guidance. *Handbook of Clinical Neurology,**173*, 25–42. 32958178 10.1016/B978-0-444-64150-2.00004-6

[CR9] Hagg, T. (2009). From neurotransmitters to neurotrophic factors to neurogenesis. *The Neuroscientist,**15*, 20–27. 19218228 10.1177/1073858408324789 PMC2722065

[CR10] Terranova, J. I., Ogawa, S. K., & Kitamura, T. (2019). Adult hippocampal neurogenesis for systems consolidation of memory. *Behavioural Brain Research,**372*, 112035. 31201874 10.1016/j.bbr.2019.112035

[CR11] Treves, A., Tashiro, A., Witter, M. P., & Moser, E. I. (2008). What is the mammalian dentate gyrus good for? *Neuroscience,**154*, 1155–1172. 18554812 10.1016/j.neuroscience.2008.04.073

[CR12] Garthe, A., Roeder, I., & Kempermann, G. (2016). Mice in an enriched environment learn more flexibly because of adult hippocampal neurogenesis. *Hippocampus,**26*, 261–271. 26311488 10.1002/hipo.22520 PMC5049654

[CR13] Anacker, C., Luna, V. M., Stevens, G. S., Millette, A., Shores, R., Jimenez, J. C., Chen, B., & Hen, R. (2018). Hippocampal neurogenesis confers stress resilience by inhibiting the ventral dentate gyrus. *Nature,**559*, 98–102. 29950730 10.1038/s41586-018-0262-4 PMC6118212

[CR14] Lieberwirth, C., Pan, Y., Liu, Y., Zhang, Z., & Wang, Z. (2016). Hippocampal adult neurogenesis: Its regulation and potential role in spatial learning and memory. *Brain Research,**1644*, 127–140. 27174001 10.1016/j.brainres.2016.05.015 PMC5064285

[CR15] Leal, G., Bramham, C. R., & Duarte, C. B. (2017). BDNF and hippocampal synaptic plasticity. *Vitamins and Hormones,**104*, 153–195. 28215294 10.1016/bs.vh.2016.10.004

[CR16] Bath, K. G., Akins, M. R., & Lee, F. S. (2012). BDNF control of adult SVZ neurogenesis. *Developmental Psychobiology,**54*, 578–589. 21432850 10.1002/dev.20546 PMC3139728

[CR17] Bekinschtein, P., Oomen, C. A., Saksida, L. M., & Bussey, T. J. (2011). Effects of environmental enrichment and voluntary exercise on neurogenesis, learning and memory, and pattern separation: BDNF as a critical variable? *Seminars in Cell & Developmental Biology,**22*, 536–542. 21767656 10.1016/j.semcdb.2011.07.002

[CR18] Kotlega, D., Peda, B., Zembron-Lacny, A., Golab-Janowska, M., & Nowacki, P. (2017). The role of brain-derived neurotrophic factor and its single nucleotide polymorphisms in stroke patients. *Neurologia i Neurochirurgia Polska,**51*, 240–246. 28291539 10.1016/j.pjnns.2017.02.008

[CR19] Wang, L., Zhang, Z., Wang, Y., Zhang, R., & Chopp, M. (2004). Treatment of stroke with erythropoietin enhances neurogenesis and angiogenesis and improves neurological function in rats. *Stroke,**35*, 1732–1737. 15178821 10.1161/01.STR.0000132196.49028.a4

[CR20] Kim, Y. R., Kim, H. N., Ahn, S. M., Choi, Y. H., Shin, H. K., & Choi, B. T. (2014). Electroacupuncture promotes post-stroke functional recovery via enhancing endogenous neurogenesis in mouse focal cerebral ischemia. *PLoS One,**9*, e90000. 24587178 10.1371/journal.pone.0090000 PMC3933702

[CR21] Ni, G. X., Liang, C., Wang, J., Duan, C. Q., Wang, P., & Wang, Y. L. (2020). Astragaloside IV improves neurobehavior and promotes hippocampal neurogenesis in MCAO rats though BDNF-TrkB signaling pathway. *Biomedicine & Pharmacotherapy,**130*, 110353. 32682983 10.1016/j.biopha.2020.110353

[CR22] Cichon, N., Saluk-Bijak, J., Gorniak, L., Przyslo, L., Bijak, M. (2020). Flavonoids as a natural enhancer of neuroplasticity—An overview of the mechanism of neurorestorative action. *Antioxidants (Basel), 9*. 10.3390/antiox9111035 PMC7690743 33114058

[CR23] Calis, Z., Mogulkoc, R., & Baltaci, A. K. (2020). The roles of flavonols/flavonoids in neurodegeneration and neuroinflammation. *Mini Reviews in Medicinal Chemistry,**20*, 1475–1488. 31288717 10.2174/1389557519666190617150051

[CR24] Zhao, Y., & Liu, S. (2021). Bioactivity of naringin and related mechanisms. *Die Pharmazie,**76*, 359–363. 34412734 10.1691/ph.2021.1504

[CR25] Gao, C., Wu, M., Du, Q., Deng, J., & Shen, J. (2022a). Naringin mediates adult hippocampal neurogenesis for antidepression via activating CREB signaling. *Front Cell Dev Biol,**10*, 731831. 35478969 10.3389/fcell.2022.731831 PMC9037031

[CR26] Caliskan, M., Mogulkoc, R., Baltaci, A. K., & Menevse, E. (2016). The effect of 3’,4’-dihydroxyflavonol on lipid peroxidation in rats with cerebral ischemia reperfusion injury. *Neurochemical Research,**41*, 1732–1740. 27017510 10.1007/s11064-016-1889-x

[CR27] Bederson, J. B., Pitts, L. H., Tsuji, M., Nishimura, M. C., Davis, R. L., & Bartkowski, H. (1986). Rat middle cerebral artery occlusion: Evaluation of the model and development of a neurologic examination. *Stroke,**17*, 472–476. 3715945 10.1161/01.str.17.3.472

[CR28] Livak, K. J., & Schmittgen, T. D. (2001). Analysis of relative gene expression data using real-time quantitative PCR and the 2(-Delta Delta C(T)) method. *Methods,**25*, 402–408. 11846609 10.1006/meth.2001.1262

[CR29] Spencer, J. P., Vauzour, D., & Rendeiro, C. (2009). Flavonoids and cognition: The molecular mechanisms underlying their behavioural effects. *Archives of Biochemistry and Biophysics,**492*, 1–9. 19822127 10.1016/j.abb.2009.10.003

[CR30] Zhang, J. C., Xu, H., Yuan, Y., Chen, J. Y., Zhang, Y. J., Lin, Y., & Yuan, S. Y. (2017). Delayed treatment with green tea polyphenol EGCG promotes neurogenesis after ischemic stroke in adult mice. *Molecular Neurobiology,**54*, 3652–3664. 27206430 10.1007/s12035-016-9924-0

[CR31] Feng, J., Chen, X., Lu, S., Li, W., Yang, D., Su, W., Wang, X., & Shen, J. (2018). Naringin attenuates cerebral ischemia-reperfusion injury through inhibiting peroxynitrite-mediated mitophagy activation. *Molecular Neurobiology,**55*, 9029–9042. 29627876 10.1007/s12035-018-1027-7

[CR32] Wang, L., Zhang, Z., & Wang, H. (2021). Naringin attenuates cerebral ischemia-reperfusion injury in rats by inhibiting endoplasmic reticulum stress. *Translational Neuroscience,**12*, 190–197. 34046215 10.1515/tnsci-2020-0170 PMC8134799

[CR33] Yang, J., Yuan, L., Wen, Y., Zhou, H., Jiang, W., Xu, D., & Wang, M. (2020). Protective effects of Naringin in cerebral infarction and its molecular mechanism. *Medical Science Monitor,**26*, e918772. 31901198 10.12659/MSM.918772 PMC6977645

[CR34] Gaur, V., Aggarwal, A., & Kumar, A. (2009). Protective effect of naringin against ischemic reperfusion cerebral injury: Possible neurobehavioral, biochemical and cellular alterations in rat brain. *European Journal of Pharmacology,**616*, 147–154. 19577560 10.1016/j.ejphar.2009.06.056

[CR35] Dunham, N. W., & Miya, T. S. (1957). A note on a simple apparatus for detecting neurological deficit in rats and mice. *Journal of the American Pharmacists Association,**46*, 208–209. 10.1002/jps.3030460322 13502156

[CR36] Lubrich, C., Giesler, P., & Kipp, M. (2022). Motor behavioral deficits in the Cuprizone model: Validity of the Rotarod test paradigm. *International Journal of Molecular Sciences,**23*(19), 11342. 36232643 10.3390/ijms231911342 PMC9570024

[CR37] Hunter, A. J., Hatcher, J., Virley, D., Nelson, P., Irving, E., Hadingham, S. J., & Parsons, A. A. (2000). Functional assessments in mice and rats after focal stroke. *Neuropharmacology,**39*, 806–816. 10699446 10.1016/s0028-3908(99)00262-2

[CR38] Tamakoshi, K., Meguro, K., Takahashi, Y., Oshimi, R., & Iwasaki, N. (2023). Comparison of motor function recovery and brain changes in intracerebral hemorrhagic and ischemic rats with similar brain damage. *NeuroReport,**34*, 332–337. 36966806 10.1097/WNR.0000000000001898

[CR39] Shi, L. F., Wang, C. J., Yu, K. W., Wu, J. F., & Zhang, Q. Q. (2023). An enriched environment promotes motor function through neuroprotection after cerebral ischemia. *BioMed Research International,**2023*, 4143633. 36817860 10.1155/2023/4143633 PMC9931462

[CR40] Altman, J., & Das, G. D. (1965). Autoradiographic and histological evidence of postnatal hippocampal neurogenesis in rats. *The Journal of Comparative Neurology,**124*, 319–335. 5861717 10.1002/cne.901240303

[CR41] Jhelum, P., Radhakrishnan, M., Paul, A. R. S., Dey, S. K., Kamle, A., Kumar, A., Sharma, A., & Chakravarty, S. (2022). Neuroprotective and proneurogenic effects of glucosamine in an internal carotid artery occlusion model of ischemia. *Neuromolecular Medicine,**24*, 268–273. 34837638 10.1007/s12017-021-08697-5

[CR42] Wiltrout, C., Lang, B., Yan, Y., Dempsey, R. J., & Vemuganti, R. (2007). Repairing brain after stroke: A review on post-ischemic neurogenesis. *Neurochemistry International,**50*, 1028–1041. 17531349 10.1016/j.neuint.2007.04.011

[CR43] Gu, W., Brannstrom, T., & Wester, P. (2000). Cortical neurogenesis in adult rats after reversible photothrombotic stroke. *Journal of Cerebral Blood Flow and Metabolism,**20*, 1166–1173. 10950377 10.1097/00004647-200008000-00002

[CR44] Bellenchi, G. C., Volpicelli, F., Piscopo, V., Perrone-Capano, C., & di Porzio, U. (2013). Adult neural stem cells: An endogenous tool to repair brain injury? *Journal of Neurochemistry,**124*, 159–167. 23134340 10.1111/jnc.12084

[CR45] Kuhn, H. G., Toda, T., & Gage, F. H. (2018). Adult hippocampal neurogenesis: A coming-of-age story. *Journal of Neuroscience,**38*, 10401–10410. 30381404 10.1523/JNEUROSCI.2144-18.2018 PMC6284110

[CR46] Kreuzberg, M., Kanov, E., Timofeev, O., Schwaninger, M., Monyer, H., & Khodosevich, K. (2010). Increased subventricular zone-derived cortical neurogenesis after ischemic lesion. *Experimental Neurology,**226*, 90–99. 20713052 10.1016/j.expneurol.2010.08.006

[CR47] Bregere, C., Fisch, U., Sailer, M. H., Lieb, W. S., Chicha, L., Goepfert, F., Kremer, T., & Guzman, R. (2017). Neonatal hypoxia-ischemia in rat increases doublecortin concentration in the cerebrospinal fluid. *European Journal of Neuroscience,**46*, 1758–1767. 28548285 10.1111/ejn.13612

[CR48] Couillard-Despres, S., Winner, B., Schaubeck, S., Aigner, R., Vroemen, M., Weidner, N., Bogdahn, U., Winkler, J., Kuhn, H. G., & Aigner, L. (2005). Doublecortin expression levels in adult brain reflect neurogenesis. *European Journal of Neuroscience,**21*, 1–14. 15654838 10.1111/j.1460-9568.2004.03813.x

[CR49] Plumpe, T., Ehninger, D., Steiner, B., Klempin, F., Jessberger, S., Brandt, M., Romer, B., Rodriguez, G. R., Kronenberg, G., & Kempermann, G. (2006). Variability of doublecortin-associated dendrite maturation in adult hippocampal neurogenesis is independent of the regulation of precursor cell proliferation. *BMC Neuroscience,**7*, 77. 17105671 10.1186/1471-2202-7-77 PMC1657022

[CR50] Mullen, R. J., Buck, C. R., & Smith, A. M. (1992). NeuN, a neuronal specific nuclear protein in vertebrates. *Development,**116*, 201–211. 1483388 10.1242/dev.116.1.201

[CR51] Duan, W., Zhang, Y. P., Hou, Z., Huang, C., Zhu, H., Zhang, C. Q., & Yin, Q. (2016). Novel insights into NeuN: From neuronal marker to splicing regulator. *Molecular Neurobiology,**53*, 1637–1647. 25680637 10.1007/s12035-015-9122-5

[CR52] Ortiz-Lopez, L., Marquez-Valadez, B., Gomez-Sanchez, A., Silva-Lucero, M. D., Torres-Perez, M., Tellez-Ballesteros, R. I., Ichwan, M., Meraz-Rios, M. A., Kempermann, G., & Ramirez-Rodriguez, G. B. (2016). Green tea compound epigallo-catechin-3-gallate (EGCG) increases neuronal survival in adult hippocampal neurogenesis in vivo and in vitro. *Neuroscience,**322*, 208–220. 26917271 10.1016/j.neuroscience.2016.02.040

[CR53] Stagni, F., Giacomini, A., Guidi, S., Emili, M., Uguagliati, B., Salvalai, M. E., Bortolotto, V., Grilli, M., Rimondini, R., & Bartesaghi, R. (2017). A flavonoid agonist of the TrkB receptor for BDNF improves hippocampal neurogenesis and hippocampus-dependent memory in the Ts65Dn mouse model of DS. *Experimental Neurology,**298*, 79–96. 28882412 10.1016/j.expneurol.2017.08.018

[CR54] Prajit, R., Sritawan, N., Suwannakot, K., Naewla, S., Aranarochana, A., Sirichoat, A., Pannangrong, W., Wigmore, P., Welbat, J. U. (2020). Chrysin protects against memory and hippocampal neurogenesis depletion in D-galactose-induced aging in rats. *Nutrients, 12*. 10.3390/nu12041100 PMC7230764 32316121

[CR55] Sawamoto, A., Okuyama, S., Yamamoto, K., Amakura, Y., Yoshimura, M., Nakajima, M., & Furukawa, Y. (2016). 3,5,6,7,8,3’,4’-Heptamethoxyflavone, a citrus flavonoid, ameliorates corticosterone-induced depression-like behavior and restores brain-derived neurotrophic factor expression, neurogenesis, and neuroplasticity in the hippocampus. *Molecules,**21*, 541. 27120588 10.3390/molecules21040541 PMC6273269

[CR56] Okuyama, S., Shimada, N., Kaji, M., Morita, M., Miyoshi, K., Minami, S., Amakura, Y., Yoshimura, M., Yoshida, T., Watanabe, S., Nakajima, M., & Furukawa, Y. (2012). Heptamethoxyflavone, a citrus flavonoid, enhances brain-derived neurotrophic factor production and neurogenesis in the hippocampus following cerebral global ischemia in mice. *Neuroscience Letters,**528*, 190–195. 22985518 10.1016/j.neulet.2012.08.079

[CR57] Okuyama, S., Morita, M., Miyoshi, K., Nishigawa, Y., Kaji, M., Sawamoto, A., Terugo, T., Toyoda, N., Makihata, N., Amakura, Y., Yoshimura, M., Nakajima, M., & Furukawa, Y. (2014). 3,5,6,7,8,3’,4’-Heptamethoxyflavone, a citrus flavonoid, on protection against memory impairment and neuronal cell death in a global cerebral ischemia mouse model. *Neurochemistry International,**70*, 30–38. 24657445 10.1016/j.neuint.2014.03.008

[CR58] Zhuang, P. W., Cui, G. Z., Zhang, Y. J., Zhang, M. X., Guo, H., Zhang, J. B., Lu, Z. Q., Isaiah, A. O., & Lin, Y. X. (2013). Baicalin regulates neuronal fate decision in neural stem/progenitor cells and stimulates hippocampal neurogenesis in adult rats. *CNS Neuroscience & Therapeutics,**19*, 154–162. 23302221 10.1111/cns.12050 PMC6493621

[CR59] Gao, H., Huang, N., Wang, W., Zhang, L., Cai, L., Chen, M., & Li, W. (2022). Astragalus flavone induces proliferation and differentiation of neural stem cells in a cerebral infarction model. *Medical Science Monitor,**28*, e933830. 35250022 10.12659/MSM.933830 PMC8915658

[CR60] Yang, W., Zhou, K., Zhou, Y., An, Y., Hu, T., Lu, J., Huang, S., & Pei, G. (2018). Naringin dihydrochalcone ameliorates cognitive deficits and neuropathology in APP/PS1 transgenic mice. *Frontiers in Aging Neuroscience,**10*, 169. 29922152 10.3389/fnagi.2018.00169 PMC5996202

[CR61] Rostamian, S., Heidari-Soureshjani, S., & Sherwin, C. M. T. (2023). The therapeutic effect of Silymarin and Silibinin on depression and anxiety disorders and possible mechanism in the brain: A systematic review. *Central Nervous System Agents in Medicinal Chemistry,**23*, 86–94. 37612866 10.2174/1871524923666230823094403

[CR62] Tang, Q., Takashima, K., Zeng, W., Okano, H., Zou, X., Takahashi, Y., Ojiro, R., Ozawa, S., Koyanagi, M., Maronpot, R. R., Yoshida, T., & Shibutani, M. (2023). Amelioration of lipopolysaccharides-induced impairment of fear memory acquisition by alpha-glycosyl isoquercitrin through suppression of neuroinflammation in rats. *Journal of Toxicological Sciences,**48*, 121–137. 36858638 10.2131/jts.48.121

[CR63] Okuyama, S., Nakashima, T., Nakamura, K., Shinoka, W., Kotani, M., Sawamoto, A., Nakajima, M., Furukawa, Y. (2018). Inhibitory effects of Auraptene and Naringin on astroglial activation, tau hyperphosphorylation, and suppression of neurogenesis in the hippocampus of streptozotocin-induced hyperglycemic mice. *Antioxidants (Basel)*, 7. 10.3390/antiox7080109 PMC6115810 30126250

[CR64] Leal-Galicia, P., Chavez-Hernandez, M. E., Mata, F., Mata-Luevanos, J., Rodriguez-Serrano, L. M., Tapia-de-Jesus, A., & Buenrostro-Jauregui, M. H. (2021). Adult neurogenesis: A story ranging from controversial new neurogenic areas and human adult neurogenesis to molecular regulation. *International Journal of Molecular Sciences,**22*(21), 11489. 34768919 10.3390/ijms222111489 PMC8584254

[CR65] Lee, J., Duan, W., & Mattson, M. P. (2002). Evidence that brain-derived neurotrophic factor is required for basal neurogenesis and mediates, in part, the enhancement of neurogenesis by dietary restriction in the hippocampus of adult mice. *Journal of Neurochemistry,**82*, 1367–1375. 12354284 10.1046/j.1471-4159.2002.01085.x

[CR66] Quesseveur, G., David, D. J., Gaillard, M. C., Pla, P., Wu, M. V., Nguyen, H. T., Nicolas, V., Auregan, G., David, I., Dranovsky, A., Hantraye, P., Hen, R., Gardier, A. M., Deglon, N., & Guiard, B. P. (2013). BDNF overexpression in mouse hippocampal astrocytes promotes local neurogenesis and elicits anxiolytic-like activities. *Translational Psychiatry,**3*, e253. 23632457 10.1038/tp.2013.30 PMC3641417

[CR67] Vitaliano, G. D., Kim, J. K., Kaufman, M. J., Adam, C. W., Zeballos, G., Shanmugavadivu, A., Subburaju, S., McLaughlin, J. P., Lukas, S. E., & Vitaliano, F. (2022). Clathrin-nanoparticles deliver BDNF to hippocampus and enhance neurogenesis, synaptogenesis and cognition in HIV/neuroAIDS mouse model. *Communications Biology,**5*, 236. 35301411 10.1038/s42003-022-03177-3 PMC8931075

[CR68] Scharfman, H., Goodman, J., Macleod, A., Phani, S., Antonelli, C., & Croll, S. (2005). Increased neurogenesis and the ectopic granule cells after intrahippocampal BDNF infusion in adult rats. *Experimental Neurology,**192*, 348–356. 15755552 10.1016/j.expneurol.2004.11.016

[CR69] Numakawa, T., Odaka, H., & Adachi, N. (2017). Actions of brain-derived neurotrophic factor and glucocorticoid stress in neurogenesis. *International Journal of Molecular Sciences,**18*(11), 2312. 29099059 10.3390/ijms18112312 PMC5713281

[CR70] Liu, W., Wang, X., O’Connor, M., Wang, G., & Han, F. (2020). Brain-derived neurotrophic factor and its potential therapeutic role in stroke comorbidities. *Neural Plasticity,**2020*, 1969482. 32399020 10.1155/2020/1969482 PMC7204205

[CR71] Zhu, Z. H., Jia, F., Ahmed, W., Zhang, G. L., Wang, H., Lin, C. Q., Chen, W. H., & Chen, L. K. (2023). Neural stem cell-derived exosome as a nano-sized carrier for BDNF delivery to a rat model of ischemic stroke. *Neural Regeneration Research,**18*, 404–409. 35900437 10.4103/1673-5374.346466 PMC9396474

[CR72] Schabitz, W. R., Steigleder, T., Cooper-Kuhn, C. M., Schwab, S., Sommer, C., Schneider, A., & Kuhn, H. G. (2007). Intravenous brain-derived neurotrophic factor enhances poststroke sensorimotor recovery and stimulates neurogenesis. *Stroke,**38*, 2165–2172. 17510456 10.1161/STROKEAHA.106.477331

[CR73] Tan, X. D., Liu, B., Jiang, Y., Yu, H. J., & Li, C. Q. (2021). Gadd45b mediates environmental enrichment-induced neurogenesis in the SVZ of rats following ischemia stroke via BDNF. *Neuroscience Letters,**745*, 135616. 33421485 10.1016/j.neulet.2020.135616

[CR74] Ahmad, M. F., Naseem, N., Rahman, I., Imam, N., Younus, H., Pandey, S. K., & Siddiqui, W. A. (2022). Naringin attenuates the diabetic neuropathy in STZ-induced type 2 diabetic Wistar rats. *Life (Basel)*, 12. 10.3390/life12122111 PMC9782177 36556476

[CR75] Wang, G., Yang, H., Zuo, W., & Mei, X. (2023). Antidepressant-like effect of acute dose of Naringin involves suppression of NR1 and activation of protein kinase A/cyclic adenosine monophosphate response element-binding protein/brain-derived neurotrophic factor signaling in hippocampus. *Behavioural Pharmacology,**34*, 101–111. 36503881 10.1097/FBP.0000000000000713

[CR76] Choi, G. Y., Kim, H. B., Hwang, E. S., Park, H. S., Cho, J. M., Ham, Y. K., Kim, J. H., Mun, M. K., Maeng, S., & Park, J. H. (2023). Naringin enhances long-term potentiation and recovers learning and memory deficits of amyloid-beta induced Alzheimer’s disease-like behavioral rat model. *Neurotoxicology,**95*, 35–45. 36549596 10.1016/j.neuro.2022.12.007

